# Hemolytic Anemia: Sneaky Cause, Leaky Valve

**DOI:** 10.7759/cureus.8370

**Published:** 2020-05-31

**Authors:** Maitreyee Rai, Muhammad Usman Ali, Charles Geller

**Affiliations:** 1 Internal Medicine, Crozer-Chester Medical Center, Upland, USA; 2 Cardiothoracic Surgery, Crozer-Chester Medical Center, Upland, USA

**Keywords:** hemolytic anemia, paravalvular leaks, mechanical mitral valve complications, anemia and hyperbilirubinemia

## Abstract

Intravascular hemolysis is a known complication of prosthetic heart valves. Severe hemolysis is rare (<1%) with the use of newer generation prosthetic valves. This usually occurs due to paravalvular leaks (PVLs). We present a case of hyperbilirubinemia and hemolytic anemia occurring as a result of a PVL of a prosthetic mechanical mitral valve. The patient was a 49-year-old female with a past medical history of rheumatic heart disease status following two mitral valve replacements each with a mechanical prosthesis; she presented with a complaint of worsening fatigue, epigastric pain, nausea, and vomiting. On examination, she had scleral icterus. Heart auscultation revealed a crisp mechanical S1 click and a soft 2/6 systolic murmur in the left lower sternal border. Her abdomen was soft with mild epigastric and right upper quadrant tenderness, and no Murphy’s sign. Her labs revealed a white blood cell count of 7.0 x 10^3^/microliter, hemoglobin 10.5 g/dL, hematocrit 29.7%, total bilirubin 6.9 mg/dL, direct bilirubin 0.8 mg/dL, alkaline phosphatase (ALP) 62 U/L, aspartate aminotransferase (AST) 79 U/L, and alanine aminotransferase (ALT) 56 U/L. An ultrasound of the abdomen revealed cholelithiasis without pericholecystic fluid collection and no ultrasonographic Murphy’s sign. Magnetic resonance cholangiopancreatography ruled out acute cholecystitis or intra- or extra-hepatic biliary ductal dilatation. A transesophageal echocardiogram showed a well-seated mitral valve prosthesis with a significant PVL and likely moderate mitral regurgitation. The patient was evaluated for possible hemolysis. Lactate dehydrogenase was 1155 U/L, haptoglobin was <30 mg/dL, and reticulocyte count was 5.2%. She underwent a mitral valve re-replacement with a mechanical prosthesis. An echocardiogram after the surgery showed the mechanical prosthesis mitral valve with no residual PVL.

## Introduction

Intravascular hemolysis occurring as a result of a prosthetic heart valve is a well-known phenomenon and is usually mild and sub-clinical. Hemolysis severe enough to cause anemia is rarely seen (<1%) with the use of newer generation prosthetic valves [1,2]. This usually occurs secondary to a paravalvular leak (PVL), which is a relatively common complication occurring in 17% of cases [1-5]. It can lead to significant morbidity depending on the severity of the leak. Anemia is believed to occur due to a combination of foreign body and shearing stress damaging the red blood cells (RBCs) resulting in hemolysis. Patients with a significant PVL may also present with heart failure and elevated cardiac filling pressure [4]. The bi-leaflet, central flow design, mechanical prosthesis has an extremely good hemodynamic profile. It is also known to be structurally durable [6].

## Case presentation

The patient was a 49-year-old female with a past medical history of rheumatic heart disease status following two mitral valve replacements each with a mechanical prosthesis. She presented to the emergency department with a complaint of worsening fatigue, shortness of breath, epigastric pain, nausea, and vomiting. The patient also reported a history of jaundice and dark urine. Other significant past medical history included sick sinus syndrome with a pacemaker in situ, hepatitis B infection, and hyperlipidemia. On physical examination, blood pressure was 157/76 mm Hg, heart rate was 77 beats per minute, temperature was 97.7°F, respiratory rate was 18/minute, and oxygen saturation was 98% on room air. Scleral icterus and conjunctival pallor were noted. Her neck was supple without masses or bruits and skin was without rashes or lesions. Cardiac auscultation was significant for a crisp mechanical S1 click with a soft 2/6 systolic murmur in the left lower sternal border. Lungs were clear to auscultation bilaterally without rhonchi, rales or wheezes. The abdomen was soft with epigastric and right upper quadrant tenderness noted; no Murphy’s sign, guarding, or rebound tenderness; and positive normoactive bowel sounds. Laboratory studies are shown in Table 1.

**Table 1:** Results for laboratory investigations WBC, white blood cell; RBC, red blood cell; HPF, high power field

Laboratory investigations [normal]	
White blood cell [4.8-10.8 x 10^3^/microliter]	7.0
Hemoglobin [11.6-15.0 g/dL]	10.5
Hematocrit [37.0%-47.0%]	29.7
Mean corpuscular volume [80.0-98.0 fL]	87.4
Mean corpuscular hemoglobin [27.0-31.0 pg]	30.9
Mean corpuscular hemoglobin concentration [31.0-37.0 g/dL]	35.4
Red cell distribution width [11.4%-14.7%]	16.9
Platelet [145-400 x 10^3^/microliter]	227
Sodium [135-146 MMOL/L]	138
Potassium [3.5-5.1 MMOL/L]	3.7
Chloride [96-106 MMOL/L]	100
Bicarbonate (CO_2_) [24-32 MMOL/L]	27
Blood urea nitrogen [10-20 mg/dL]	21
Creatinine [0.6-1.1 mg/dL]	1.0
Bilirubin, total [0.3-1.0 mg/dL]	6.9
Bilirubin, direct [0.0-0.2 mg/dL]	0.8
Alkaline phosphatase [30-120 U/L]	56
Aspartate aminotransferase [5-27 U/L]	78
Alanine transaminase [7-52 U/L]	58
Troponin [<0.04 ng/mL]	Non-detectable
Erythrocyte sedimentation rate [0-20 mm/hr]	8
C-reactive protein [<5.0 mg/L]	5.2
Urine analysis	
pH [5.0-8.0]	5.0
Color, clarity	Amber, hazy
Glucose [(negative) mg/dL]	Negative
Bilirubin [(negative) mg/dL)	Negative
Urobilinogen [(negative) mg/dL]	2.0
Nitrates [negative]	Negative
Blood [(negative)/mL]	Moderate
RBC [0-2/HPF]	6-10
WBC [0-2/HPF]	20-30
Leukocyte esterase	Large
Bacteria	Rare
Urine culture	No growth
Blood culture	No growth

Electrocardiogram (EKG) and chest x-ray were within normal limits. Computed tomography angiogram of the chest revealed no pulmonary embolism, thoracic aortic aneurysm, or dissection. Abdominal ultrasound showed cholelithiasis with pericholecystic fluid, but no gallbladder wall thickening or focal tenderness. Common bile duct diameter was measured as 4 mm with no intrahepatic bile duct dilation.

**Figure 1:**
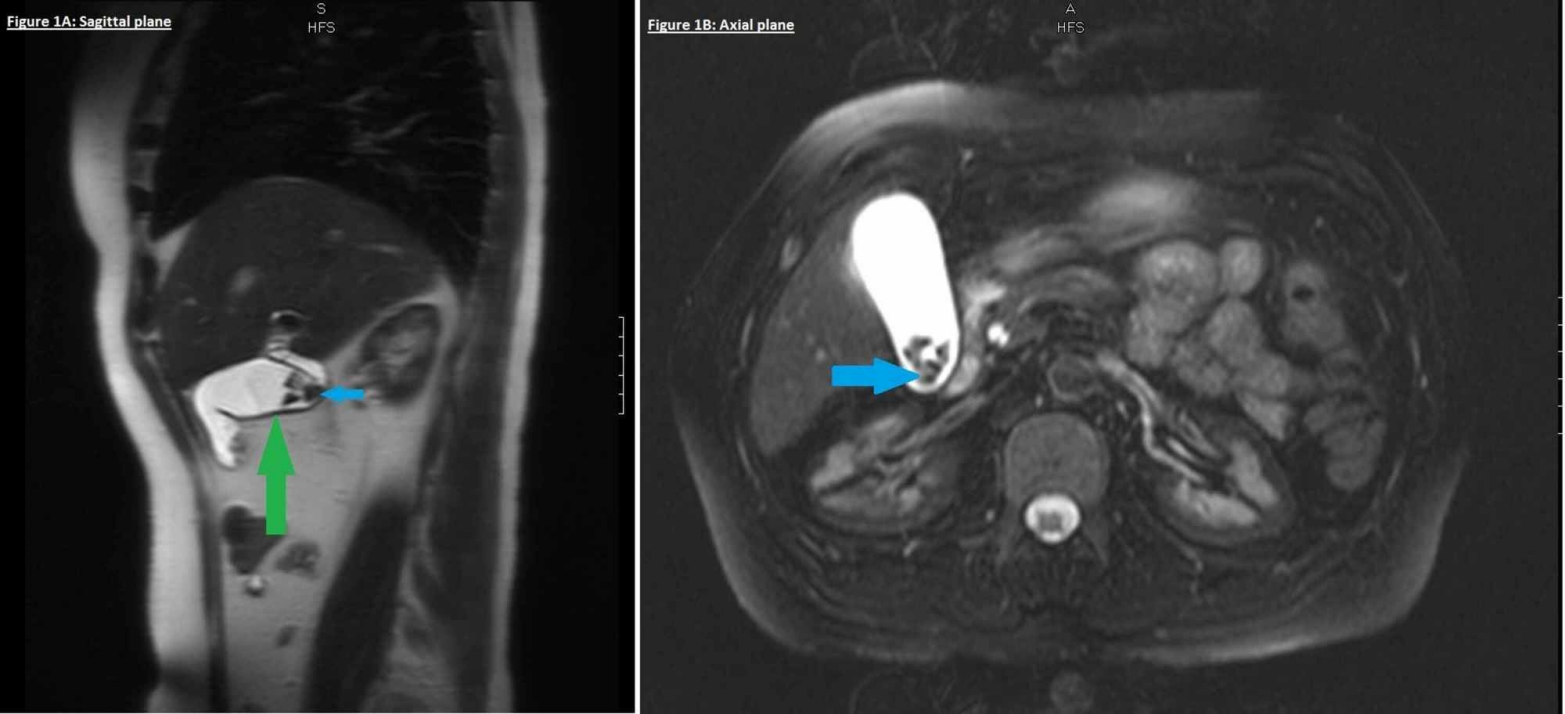
MRCP (sagittal and axial planes) revealing cholelithiasis (blue arrows) with mild gallbladder wall thickening (green arrow) indicative of chronic cholecystitis MRCP, magnetic resonance cholangiopancreatography

**Figure 2:**
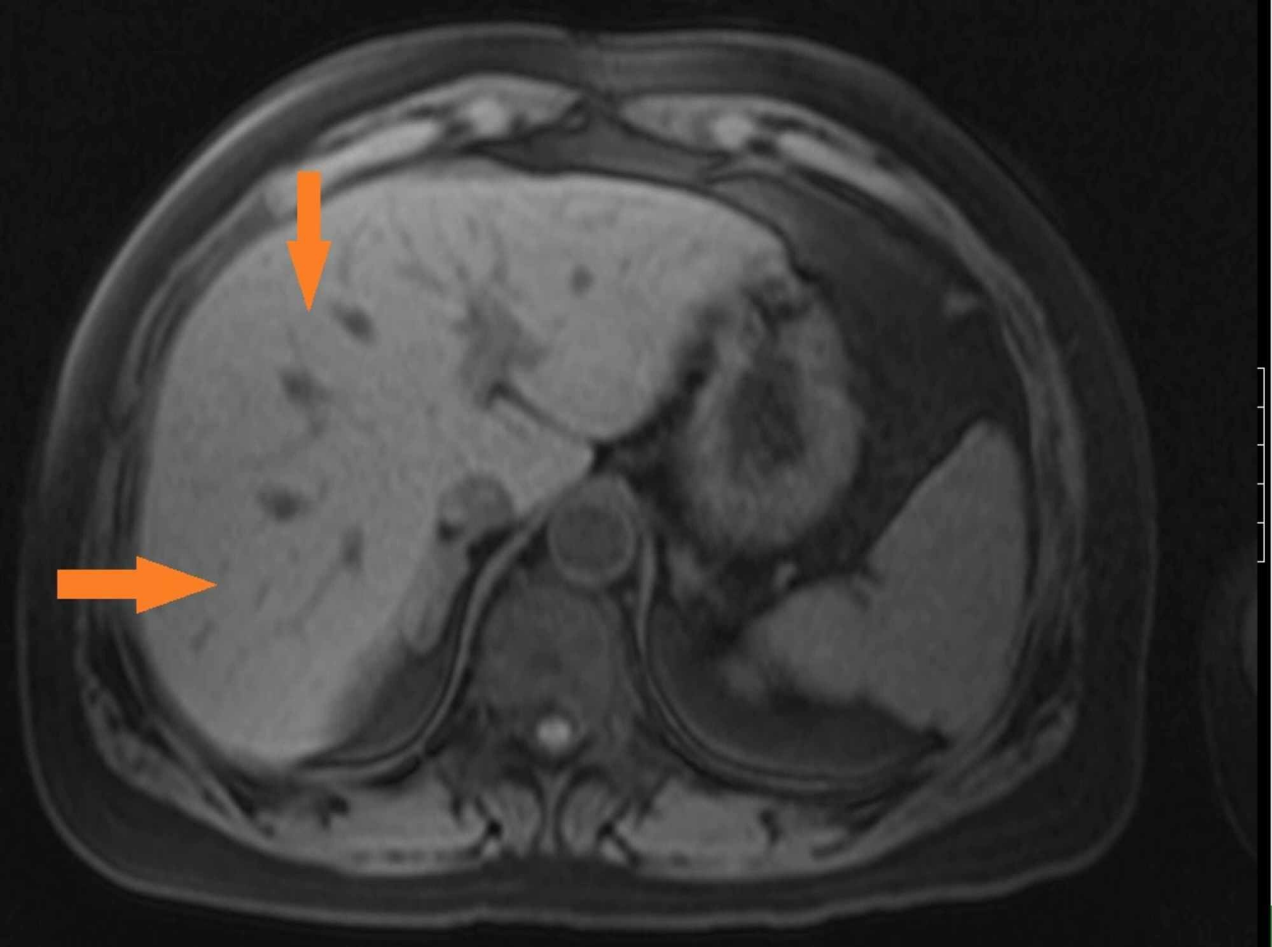
MRCP showing mild hepatomegaly with hepatic steatosis (orange arrows) MRCP, magnetic resonance cholangiopancreatography

The patient had a history of elevated liver function tests in a hepatocellular pattern. She also had a history of positive hepatitis B surface antigen. A total bilirubin of 6.9 mg/dL was higher than her usual baseline. A magnetic resonance cholangiopancreatography (MRCP) of the abdomen was unremarkable except for chronic cholecystitis (Figures 1, 2).

A hepatitis panel was also checked along with other viral antibodies (Table 2). The findings were not consistent with a reactivation of hepatitis B infection. 

**Table 2:** Hepatitis panel and other viral investigations IgM, immunoglobulin M

Viral investigations	
Hepatitis B core antibody, total	Reactive
Hepatitis B core IgM	Non-reactive
Hepatitis B surface antigen	Reactive
Hepatitis B E antigen	Non-reactive
Hepatitis C antibody	Non-reactive
Epstein-Barr virus IgM antibody	Non-reactive
Cytomegalovirus IgM antibody (<1.10)	0.29 (non-reactive)
Human immunodeficiency virus	Non-reactive

**Figure 3:**
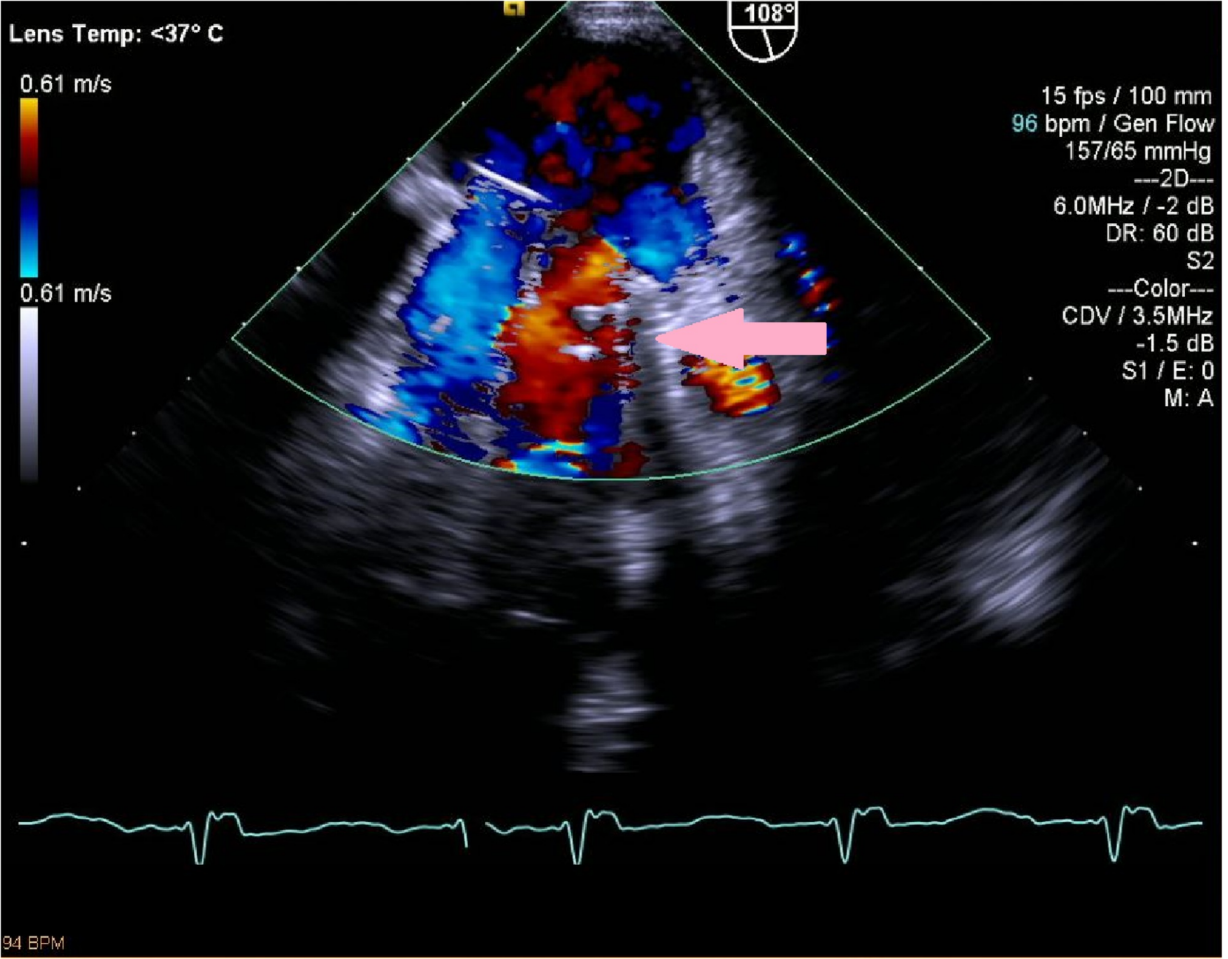
TEE image showing paravalvular leak (pink arrow) TEE, transesophageal echocardiogram

Given the patient's significant cardiac history, a trans-thoracic echocardiogram (TTE) was performed that revealed mildly depressed biventricular systolic function (left ventricular ejection fraction 45%-50%), prosthetic mitral valve well seated, but at least mild paravalvular insufficiency. Subsequently, a transesophageal echocardiogram (TEE) showed normal left ventricular systolic function (ejection fraction 55%), a well-seated mitral valve prosthesis with a significant PVL and likely moderate mitral regurgitation with some pulmonary flow reversal, and no pericardial effusion (Figure 3).

Cardiac catheterization revealed normal coronary arteries, mild left ventricular systolic dysfunction, mitral regurgitation secondary to multiple PVLs, and normal cardiac output/index.

Given these findings, hemolytic anemia was suspected. This was further evaluated with a hemolysis panel (Table 3).

**Table 3:** Tests for anemia evaluation

Anemia investigations [normal]	
Lactate dehydrogenase [140-271 U/L]	1155
Haptoglobin [44-215 mg/dL]	<30
Reticulocyte count [0.0%-2.0%]	5.2
Protein, total [6.5-8.0 g/dL]	6.7
Albumin [3.4-5.0 g/dL]	4.1
Alpha 1 globulin [0.2-0.3 g/dL]	0.30
Alpha 2 globulin [0.5-0.9 g/dL]	0.50
Gamma globulin [0.8-1.7 g/dL]	0.90
Serum protein electrophoresis	Normal electrophoretic pattern
Immunoglobulin G [681-1648 mg/dL]	954
Immunoglobulin A [87-474 mg/dL]	170
Immunoglobulin M [48-312 mg/dL]	73
Beta 1 globulin [0.4-0.6 g/dL]	0
Beta 2 globulin [0.2-0.5 g/dL]	0
Kappa light chains, total [176-443 mg/dL]	247
Lambda light chains, total [91-240 mg/dL]	107
Kappa/lambda light chains ratio (K/L) [1.29 to 2.55]	2.0

These findings were suggestive of hemolytic anemia. The differential diagnosis was of hemolysis occurring either secondary to mechanical shearing due to valvular pathology or an autoimmune etiology. Serum protein electrophoresis, immunoglobulin levels, as shown in Table 3, and direct Coombs test were unremarkable, eliminating autoimmune etiology as the cause for hemolysis. Cardiothoracic surgeons did a third time re-operation, a right mini-thoracotomy Heartport mitral valve re-replacement with a mechanical valve was performed without complications. A post-operative TEE demonstrated a normally functioning prosthesis with no PVL, and low normal left ventricular systolic function (ejection fraction 50%). The hyperbilirubinemia resolved, with a total bilirubin of 0.9 mg/dL at discharge. Hemoglobin improved to 12.7 g/dL at the 6-month follow-up.

## Discussion

Our patient was diagnosed with a prosthetic PVL as the etiology of her hemolytic anemia and hyperbilirubinemia. While a gastroenterology workup is warranted in all patients with hyperbilirubinemia, this case highlights the importance of maintaining a broad differential diagnosis. The multidisciplinary management of hemolytic anemia is essential.

Hemolytic anemia is a result of increased and premature RBC destruction. This can be either acute or chronic, inherited or acquired. The causes for destruction may include intracorpuscular or extracorpuscular defect. Diagnosis and workup starts with a thorough history including an emphasis on the onset of symptoms of anemia, bleeding history, predisposing risk factors for hemolysis such as familial or inherited disorders, history of blood transfusion, infections, or initiation of new medications [7]. The physical exam may reveal signs of pallor, jaundice, cardiac murmurs (for valvular pathologies), or splenomegaly, and help to further narrow the possible causes and streamline a diagnostic workup. Laboratory studies can show low hemoglobin, hematocrit, and RBC count with an elevated reticulocyte count as well as findings consistent with increased RBC destruction like elevated lactate dehydrogenase, indirect hyperbilirubinemia, and low haptoglobin levels. Given our patient's significant cardiac history, it was prudent to evaluate the possibility of prosthetic valve dysfunction. Other causes of hemolysis such as inherited disorders, drug-induced, transfusion-related, disseminated intravascular coagulation or hemolytic uremic syndrome seemed unlikely based on history and initial studies. The patient's workup for autoimmune causes of hemolysis was negative.

PVL is considered a complication of surgical cardiac valve replacement and usually occurs early although our patient had a later presentation. This can happen due to incomplete approximation of the prosthesis to the valve annulus resulting in periprosthetic regurgitation. The actual incidence of PVL cannot be estimated accurately and differs across different registries. It has been noted that paravalvular mitral leaks (PVMLs) are more common (7%-17%) than paravalvular aortic leaks (2%-10%) [8]. The clinical presentation of PVLs is highly variable and depends on the severity of the leak. Mild PVLs may be hemodynamically insignificant and patients are therefore asymptomatic. With severe PVLs, findings of congestive heart failure and hemolytic anemia are possible. The first indication of a PVL can be the presence of an abnormal murmur on cardiac auscultation. However, it may be too soft to be appreciated and is highly dependent on the skill and expertise of the examining physician. Cardiac imaging studies, therefore, are the preferred way to evaluate prosthetic valve function with echocardiography being the gold standard. TTE is typically done initially and can give information on cardiac function, chambers, and valves, as well potentially identify PVLs using color Doppler. However, prosthetic valves tend to produce acoustic artifacts masking the presence or severity of the jet. Therefore, it is imperative that TTE be followed by the more confirmatory TEE in cases where PVL is suspected clinically [8]. A newer diagnostic approach, three-dimensional (3D) TEE has allowed even better visualization of the shape and size of PVLs [9].

For patients with symptomatic and/or significant PVLs, surgical repair is the traditional treatment approach of choice. The surgical approach has shown to significantly improve patient outcomes at 1, 5, and 10 years compared to medical treatment alone [10]. Percutaneous repair of a PVML is another option; however, it has technical limitations associated with the approach (trans-septal, trans-apical); cardiac anatomy; and location, size, and number of the defects [4].

## Conclusions

While a gastroenterology workup is warranted in all patients with hyperbilirubinemia, this case highlights the importance of maintaining a broad differential including unusual mechanical causes. The hemolytic anemia should always be approached with multidisciplinary care, and paravalvular leak, even though a rare cause, needs to be ruled out. The analysis should be directly based on a patient's history and physical examination. An extensive cardiac evaluation allowed this patient to receive early corrective surgery and avoid potential future morbidities. It is worth noting that TTE plays a limited role in the assessment of a PVML secondary to acoustic masking by the prosthesis. It is instead better visualized by TEE using a probe positioned directly behind the left atrium and correlated with cardiac catheterization findings.
